# Differential diagnoses of the pale/white/atrophic disc

**Published:** 2017-03-03

**Authors:** Vivian B Osaguona

**Affiliations:** 1 Senior lecturer/consultant ophthalmologist (neuro-ophthalmology & general ophthalmology) University of Benin Teaching Hospital Benin City, Edo State, Nigeria.

**Optic atrophy, pallor of the optic nerve head, is a sign found in patients with visual loss due to pathology of the optic nerve or retinal ganglion cells. There are many causes. This article discusses the differential diagnosis of optic atrophy.**

**Figure:**
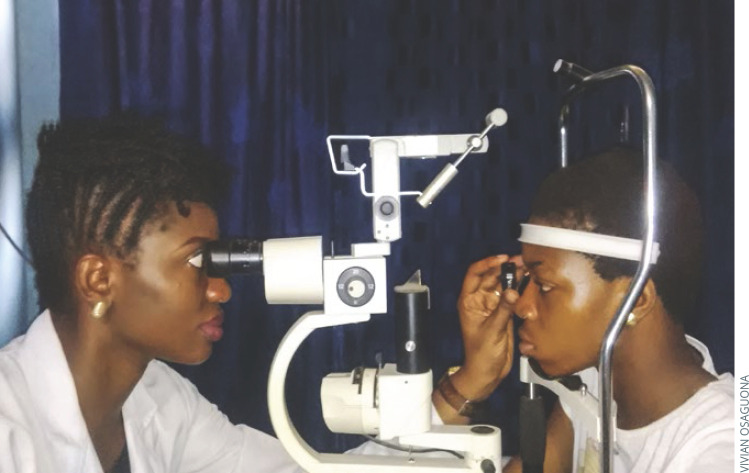
Examining the optic disc with a 90D lens at the slit lamp. NIGERIA

Optic atrophy is not a disease in itself but a clinical sign. It refers to pallor of the optic disc which results from irreversible damage to the retinal ganglion cells and axons. The axons of the retinal ganglion cells make up the optic nerve and continue onto the optic chiasm, optic tract and up to the lateral geniculate body before they synapse. Injury to the retinal ganglion cells and axons anywhere along their course from the retina to the lateral geniculate body may result in optic atrophy.

The causes of optic atrophy are numerous; they include:

1. Inflammation
2. Ischaemia
3. Compression, including raised intracranial pressure
4. Nutritional deficiencies / effect of toxins, including epidemic
5. Trauma
6. Hereditary conditions and childhood optic atrophy

**Clinical features in optic atrophy**

- Diminution of vision (central acuity/colour vision/visual field defects)
- Afferent pupil defect
- Optic disc pallor
- Reduced number of small blood vessels on the disc surface (Kestenbaum sign)
- Attenuation (thinning) of blood vessels around the disc
- Thinning of the retinal nerve fiber layer.

**Causes of pseudo optic atrophy**

**Non pathologic causes of a pale disc**

- Disc is examined with very bright light
- Large physiologic cup in axial myopia
- Post-cataract extraction

**Other (non optic atrophy) causes of a pale disc**

- Myelinated retinal nerve fibers
- Optic disc coloboma
- Optic disc hypoplasia

Clinically, optic atrophy is associated with a decrease in visual acuity and visual field defect (see upper box, left).

There are other causes of disc pallor which are not due to optic atrophy (see lower box, left) which should be excluded.

## Ophthalmoscopic classification

Optic atrophy can be classified into primary, secondary and consecutive optic atrophy. Each has characteristic features which may help to differentiate between them (**Table 1** on page 70).

**Figure 1.**
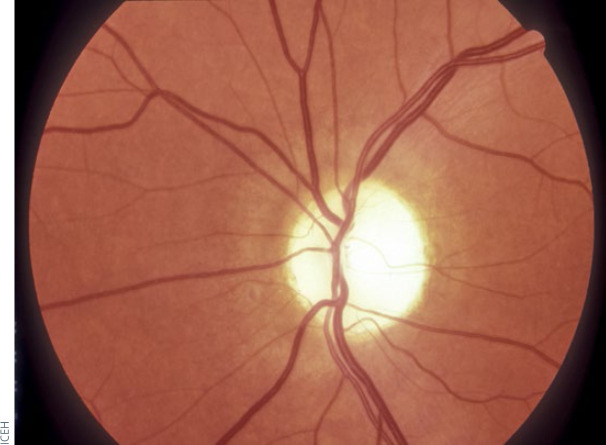
Primary optic atrophy

**Figure 2.**
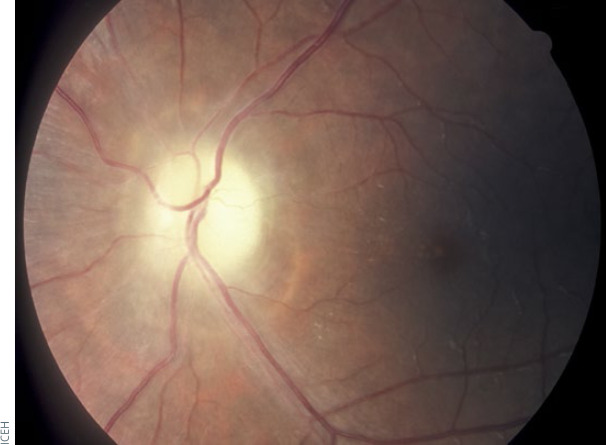
Secondary optic atrophy

**Figure 3.**
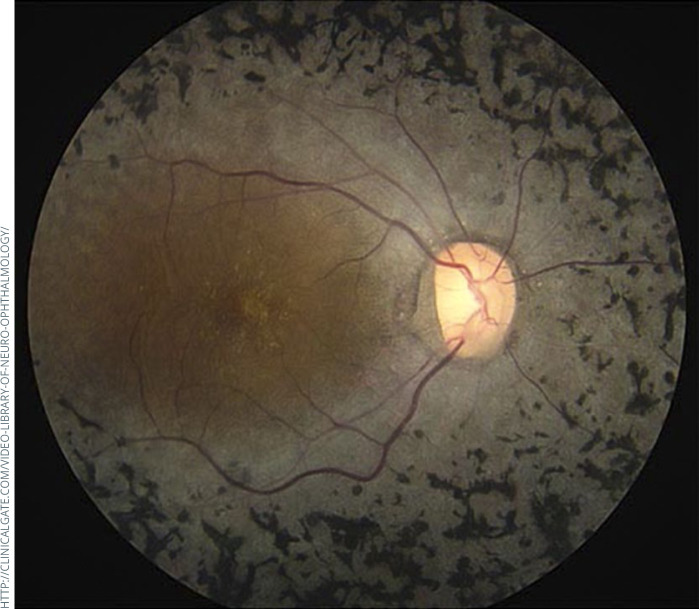
Consecutive optic atrophy (with retinitis pigmentosa)

In **primary optic atrophy** (**Figure 1** on page 71) there is no previous swelling of the optic disc. The disc is white, the margins are distinct and the retinal blood vessels at the optic nerve head appear normal. **Secondary optic atrophy** (**Figure 2** on page 71) is a consequence of long-standing swelling of the optic disc, which may be due to inflammation, ischaemia or raised intracranial pressure. The disc is greyish in colour and the margins are blurred. There is fibrosis (gliosis) of the optic nerve head and the blood vessels may appear indistinct or narrowed. **Consecutive optic atrophy** (**Figure 3** on page 72) results from chorioretinal disease such as retinitis pigmentosa or toxoplasmosis chorioretinitis. The cause of consecutive optic atrophy is usually obvious from the retinal appearance and so will not be discussed further.

Glaucoma is an important cause of optic atrophy. In glaucoma there is characteristic pathological cupping of the optic disc which together with the typical visual field loss distinguishes glaucoma from other causes of optic atrophy. Glaucoma will also not be considered further in this article. (*For articles on glaucoma please see CEHJ* Comm Eye Health Vol. 25 No. 79 & 80 2012).

**Table 1:** Ophthalmoscopic classification of optic atrophy

Sign	Primary	Secondary	Consecutive
Previous swelling of the optic disc	No	Preceded by long-standing swelling of the optic disc.	No
Disc colour	White: diffuse or sectoral pallor.	Grey	Waxy pale
Disc margins	Distinct	Blurred	Normal, attenuated arteries
Fibrosis (gliosis) of the disc	None	Gliosis of the optic nerve head	None
Cause	compression of the optic nerve or chiasm hereditary optic neuropathy nutritional optic atrophy	chronic papilloedema papillitis anterior ischaemic optic neuropathy	chorioretinal disease e.g. retinitis pigmentosa central retinal artery occlusion

## Differential diagnosis of optic atrophy

Careful evaluation of the optic disc pallor together with other clinical features and a careful history can give clues to the underlying cause. The cause of optic atrophy may be a threat to the patient's life or vision and identification of the cause may save the patient's life or vision.

## How to examine the optic disc

The optic disc can be examined using a direct ophthalmoscope or at the slit-lamp using a 70D or 90D lens. Special attention should be paid to the colour of the disc, and whether the whole disc is paler than usual, or only a segment. The edge of the disc should be examined to see whether it is distinct or indistinct, and lastly the retinal blood vessels should be examined as they course over the optic nerve head to see if they are distinct and of normal and regular thickness. Lastly, it is important to assess whether both optic discs are equally affected, or whether one disc is normal, less affected or is swollen.

## Other ocular assessment in patients with optic atrophy

As well as examing the optic disc the visual acuity should be measured and the visual fields assessed by perimetry. The pupils should be examined for response to light and colour vision should also be assessed.

Having established that optic atrophy is present, try and decide whether it is primary or secondary, as this will guide you on what questions to ask the patient as the causes can be quite different.

## History

For all patients with optic atrophy it is important to ask how long ago they noticed the loss of vision and whether it came on suddenly or gradually. More specific questions should then be asked for primary and for secondary optic atrophy (see across).

## Primary optic atrophy

Questions to ask the patient:

- Are they taking any medication for tuberculosis?
- Have they had a diagnosis of a sexually transmitted disease e.g. syphilis or HIV?
- Do they frequently take quinine for malaria?
- Do they smoke and / or drink alcohol, have they consumed methanol?
- Does their occupation entail working with chemicals?
- Do they eat cassava and if so how is it prepared?

## Secondary optic atrophy

Questions to ask the patient:

- Have they been suffering from headaches, and if so is the headache worse in the mornings?
- Is the headache accompanied by nausea or vomiting?

If the answer to these questions is yes, urgent referral is required to rule out raised intracranial pressure which may be due to a tumour.

- Have they noticed any pain behind the eye on eye movement?
- Have they had episodes of double vision, or weakness or tingling in their arms or legs?

If the answer is yes to these questions then the optic atrophy, which is often unilateral initially, may be due to multiple sclerosis and the patient should be referred to a neurologist.

- Have they had any pain in other areas of their face?

If yes, they should be referred toan ENT specialist to rule out sinus disease.

If the loss of vision was sudden, the following signs and symptons are suggestive of giant cell arteritis:

- Scalp tenderness
- Pain in the jaws when chewing
- Muscle and joint pains
- Weight loss

The clinical diagnosis is confirmed by an urgent temporal artery biopsy and high dose systemic steroids are required. Once the diagnosis has been made these patients are best managed by a physician.

## 1 Inflammatory optic neuropathy

In inflammatory disease of the optic nerve the initial appearance may be one of a swollen disc due to papillitis which over the course of a few months becomes atrophie. The visual loss may be sudden or gradual, unilateral or bilateral and may be associated with pain on eye movements.

Inflammatory optic neuropathy due to multiple sclerosis may present as a history of unilateral blurring of vision associated with pain on eye movements in a person aged 20–40 years, associated with other neurologic features such as diplopia, paraesthesia, loss of muscle power and loss of sphincter control. Neuromyelitis optica (Devic's disease) is severe and rare and presents acutely as bilateral optic neuritis with paralysis due to a transverse myelitis.

Other causes of inflammatory optic neuropathy Include tuberculosis or syphilis; and it can also occur In the presence of systemic lupus erythematosus, or sarcoidosis; occasionally it is associated with orbital or sinus infection.

## 2 Ischaemic (vascular) optic neuropathy

Optic neuropathy due to a compromised blood supply of the optic nerve usually presents as a sudden loss of vision. The optic disc pallor may be diffuse or segmental (sectoral). Segmental pallor occurs if part of the blood supply to the optic nerve is occluded, and It will be associated with an appropriate altitudinal field defect.

An important treatable cause of ischaemic optic neuropathy is giant cell arteritis (GCA). It may present with sudden acute visual loss associated with scalp tenderness, jaw pain on chewing, muscle aches and weight loss, usually in people aged over 60 years. Diagnosis is suggested by tenderness over an easily palpable temporal artery and confirmed histologically by a temporal artery biopsy. Often the ESR is highly raised. If GCA is the suspected diagnosis, treatment with high dose systemic steroids should be started urgently in order to reduce the risk of vision loss.

Anterior ischaemic optic neuropathy which is not due to arteritis may also occur in people (often elderly) with vascular risk factors such as diabetes mellitus, hypertension, hyperchlesterolaemia and/or smoking. It presents as one or more episodes of acute visual loss due to vessel occlusion at the optic nerve head. Treatment is that of the cause, however one should be careful about lowering the blood pressure when the optic nerve already has evidence of poor perfusion.

Sometimes acute visual loss may follow surgery (cardiac, neck or spine surgery), or massive blood loss due to ischaemia of the blood supply posterior to the optic nerve head.

## 3 Compressive optic neuropathy

Compressive lesions commonly present with unilateral or bilateral gradual progressive vision loss. Lesions around the orbital apex, superior orbital fissure or cavernous sinus may also present with limitation of extraocular motility from involvement of the cranial nerves 3rd, 4th and 6th.

If the optic nerve is affected there maybe a central or caecocentral field defect with an afferent pupil defect while optic chiasmal lesions which affect the decussating nasal fibres cause a bitemporal hemianopia (loss of vision in the temporal visual fields of the two eyes).

Compression of the optic nerve maybe due to a meningioma, orbital tumour, thyroid eye disease or intracranial tumours such as pituitary adenoma or carotid aneurysm.

### Raised Intracranial pressure – papilloedema and secondary atrophy

Papilloedema refers to optic disc swelling as a result of raised intracranial pressure and it is usually bilateral. There may be diminution in vision and other features of raised intracranial pressure such as headache, seizures, nausea and vomiting. Initially there is a swollen disc (papilloedema) followed later by secondary optic atrophy.

Intracranial pressure rise may result from many causes including intracranial space occupying lesions, idiopathic intracranial hypertension and meningitis. Long-standing papilloedema results in optic atrophy.

## 4 Toxic/nutritional optic neuropathy

Toxic/nutritional optic neuropathies present with bilateral, painless, symmetric vision loss which is gradual and progressive. The neuropathy typically affects the papillomacular bundle causing temporal pallor of the optic disc (the papillomacular bundle Inserts into the temporal optic disc). The visual fields show central or centrocaecal field defects.

It is important to take a history to rule out medications that are toxic to the anterior visual pathway such as ethambutol, isoniazid or sildenafil. The patient's occupation may also expose him or her to toxic substances such as lead in paints, antifreeze agents or cyanide from improper processing of cassava. A monotous diet of cassava products with little protein has been associated with optic atrophy in the “tropical amblyopia syndrome”. Deficiency of vitamin B12, B1 (thiamine) and folate can result in optic atrophy and may be seen in persons who are heavy cigarette smokers, consume alcohol and have a poor diet. Accidental ingestion of methanol (methyl alcohol) may be fatal and may cause rapid and severe visual loss.

### Epidemics of Optic Atrophy

There have been some reports of optic neuropathy affecting large numbers of people over a few months most notably in Cuba, Tanzania and Sierra Leone. Those affected are often adolescents who develop optic atrophy, sometimes with other neurological signs. The optic atrophy can be mild and limited to the temporal aspect of the disc. The cause is not fully understood, but is thought to be due to nutritional deficiencies and/or toxic effects. Those affected should be treated with multi-vitamins, including the B group, and given advice about a healthy diet.

## 5 Ocular trauma to the optic nerve

Trauma to the optic nerve may result from indirect Injury (blunt trauma) to the head or from direct injury from bony fragments or bullets, or from compression by haematoma within the orbit or optic nerve sheath. A previous history of head trauma with visual loss may be obtained.

## 6 Hereditary causes and optic atrophy in children

Children with unexplained visual loss and/or unilateral or bilateral optic atrophy should always be referred for further investigation, as an underlying cause should always be sought. Optic atrophy in children may be due to genetic factors (e.g. Leber's hereditary optic atrophy), but it might also signify that something is pressing on the nerve, or there is raised Intracranial pressure from a brain tumour which may be benign or malignant.

### Investigations

Appropriate investigations may include: blood pressure; blood glucose; erythrocyte sedimentation rate; blood test for vitamin B12, and red blood cell folate for those suspected of having nutritional optic atrophy. Chest X-ray is indicated if there are associated respiratory symptoms.

Neuroimaging of the orbit and the brain with attention to the course of the optic nerve, optic chiasm and optic tract may reveal the cause of the optic atrophy in compressive lesions.

### Management

There is no specific treatment for optic atrophy itself. The underlying cause whether inflammatory, ischaemic, compressive or metabolic should be treated if known. If there is a causative medication or toxin it should be avoided while vitamin deficiencies should be replaced. Patients with low vision may benefit from low vision devices.

## Summary

Optic atrophy is not a disease but a clinical sign. It refers to pallor of the optic disc which results from irreversible damage to fibers of the anterior visual pathway. The causes of optic atrophy are numerous, some of which may be life or sight threatening. A detailed clinical evaluation is helpful in the differential diagnosis and management of optic atrophy.
